# Regeneration during Obesity: An Impaired Homeostasis

**DOI:** 10.3390/ani10122344

**Published:** 2020-12-09

**Authors:** Abdelaziz Ghanemi, Mayumi Yoshioka, Jonny St-Amand

**Affiliations:** 1 Department of Molecular Medicine, Faculty of Medicine, Laval University, Quebec City, QC G1V 0A6, Canada; abdelaziz.ghanemi@crchudequebec.ulaval.ca; 2 Functional Genomics Laboratory, Endocrinology and Nephrology Axis, CHU de Québec-Université Laval Research Center, Quebec City, QC G1V 4G2, Canada; mayumi.yoshioka@crchudequebec.ulaval.ca

**Keywords:** obesity, regeneration, homeostasis

## Abstract

**Simple Summary:**

Regeneration represents the biological processes that allow cells and tissues to renew and develop. During obesity, a variety of changes and reactions are seen. This includes inflammation and metabolic disorders. These obesity-induced changes do impact the regeneration processes. Such impacts that obesity has on regeneration would affect tissues and organs development and would also have consequences on the outcomes of therapies that depend on cells regeneration (such as burns, radiotherapy and leukemia) given to patients suffering from obesity. Therefore, a particular attention should be given to patients suffering from obesity in biological, therapeutic and clinical contexts that depend on regeneration ability.

**Abstract:**

Obesity is a health problem that, in addition to the known morbidities, induces the generation of a biological environment with negative impacts on regeneration. Indeed, factors like DNA damages, oxidative stress and inflammation would impair the stem cell functions, in addition to some metabolic and development patterns. At the cellular and tissulaire levels, this has consequences on growth, renewal and restoration which results into an impaired regeneration. This impaired homeostasis concerns also key metabolic tissues including muscles and liver which would worsen the energy balance outcome towards further development of obesity. Such impacts of obesity on regeneration shows the need of a specific care given to obese patients recovering from diseases or conditions requiring regeneration such as burns, radiotherapy and leukemia. On the other hand, since stem cells are suggested to manage obesity, this impaired regeneration homeostasis needs to be considered towards more optimized stem cells-based obesity therapies within the context of precision medicine.

Endogenous processes and biological homeostasis require a proper regeneration ability summarized in the biological capacity to restore, renew and grow different cells and tissues. This regeneration ability (governed by complex cellular functions and molecular processes) requires optimum conditions defined by biological environments in terms of pH, cytokines, growth factors and diverse signals and messenger activities.

Within this context, obesity, as a pathological status of broken homeostasis [1,2], induces the generation of a negative regeneration environment, which, biologically, means conditions that limit or reduce the ability of cellular and tissulaire restoration, renewal and growth. Indeed, diverse works throughout the literature report direct and indirect impacts of obesity on regeneration in different cells and tissues. Stem cells are key players for regeneration and these cells are impacted by obesity. For instance, data indicate that stem cells-based hematopoietic and osteogenic regeneration is impaired during obesity [3], probably as a consequence of bone marrow adipose tissue expansion that leads to a deterioration within the skeleton [4], the obesity-induced bone marrow microenvironment modifications [5] and the increased endoplasmic reticulum stress in bone marrow mesenchymal stem cells [6]. In addition, proliferative and migratory abilities of adipose-derived mesenchymal stem cells derived from obese subjects are reduced [7]. Obesity also alters the stem cells’ differentiation potential [8]. These obesity impacts on stem cells might result from different mechanisms, including the reshape of stem cell extracellular vesicles [9], inflammation promotion [5] and the resulting surrounding microenvironment modifications resulting from obesity [10,11]. Within this context, it is worth mentioning that during obesity, inflammatory-related balance is impaired. Indeed, M1 macrophages (promote pro-inflammation) are enhanced and M2 macrophages (anti-inflammatory factor) are down-regulated [12]. Such macrophage polarization supports the classification of obesity as a chronic pro-inflammatory disease [12] leading to metabolic dysfunctions, including insulin resistance [13]. To further understand the critical balanced roles of inflammation, it is essential to state that although obesity-induced inflammation might impair regeneration, inflammatory-related processes such as those mediated by macrophages are required for damaged tissue elimination as a step preparing the regeneration as explored by Varga T et al. [14,15,16].

Regarding skeletal muscles, which form the locomotor system with the skeleton, their regeneration is also impaired by obesity [17]. Importantly, satellite cells (muscle stem cells) are reduced in obese mice (post trauma [18]) with a diminished fusion capacity [19], showing the deep impacts of obesity on muscle regeneration starting at the stem cell level. Moreover, this obesity senescence has also been said to affect satellite cells and skeletal muscle in the context of obesity [20], which indicates the worsening effect ageing has on regeneration.

Within the metabolics of skeletal muscle, the expression of the mitochondrial glycerol 3-phosphate dehydrogenase (which promotes skeletal muscle regeneration) is reduced in animal models of obesity [21]. Such mitochondrial implications are in correlation with the suggested regulatory role mitochondrial biogenesis plays during muscle regeneration [22,23]. These illustrations of the close links between obesity, mitochondria and skeletal muscle provide a piece, among others [24,25,26], of the mechanistic puzzle of obesity impacts on skeletal muscle regeneration from a metabolic perspective. Furthermore, the deficiency of vitamin D, which plays a role in skeletal muscle regeneration [27], is associated with obesity [28] as well.

The other important element linking obesity to the generation of an environment leading to reduced regeneration ability is inflammation detected within different tissues, including skeletal muscle [29], adipose tissue [30,31] and liver [30], which are the key metabolic tissues governing most of the energy balance homeostasis. Inflammation impairs the regeneration of the skeletal muscle [32], and these impacts, among others, that obesity has on muscle regeneration are of particular importance since muscle is the key tissue for energy expenditure. Therefore, reduced metabolic performance of the muscle would reduce the energy expenditure and worsen obesity by shifting the metabolic balance towards further energy storage. Importantly, the impact obesity has on skeletal muscle in terms of regeneration could worsen sarcopenic obesity and explain a part of its pathogenesis [33], including that shared with obesity and leading to commune outcomes such insulin resistance and cardiometabolic impacts [34], through reducing muscle regeneration in an obesity-developing environment. Exploring an example from liver’s regenerative properties, the other key metabolic organ, indicates that hepatic steatosis (which develops during obesity [35,36]) impairs liver regeneration through diverse mechanisms, including oxidative stress [37], which could worsen the existent energy balance status as well.

Selected growth and metabolic factors are also modified during obesity. For instance, insulin [38] and leptin [39] increase during obesity. Since such factors are related to cellular growth and metabolism [40,41,42,43,44], these changes could impact regeneration ability. Indeed, insulin roles within both adipocytes and bone stromal stem cells of the bone marrow [45] suggest an impact of increased insulin on both regeneration and metabolism, as illustrated by the obesity-associated hypermetabolism leading to bone fragility [46], especially with the important roles that marrow adipocytes play in bone homeostasis [47] and remodeling [48]. This fits with the findings that obese db/db (mice lacking leptin receptor) have their postnatal regenerative osteogenesis compromised [49]. More extensive investigations linking growth and homeostatic factors (hormones, neurotransmitters, metabolic mediators, etc.) changes during obesity to regeneration impairment would provide molecular links allowing better clarification of the mechanistic pathways and identify molecular targets to correct these effects on regeneration homeostasis.

In addition, obesity represents a risk factor for cancer [50], which represents a pathological cellular replication and therefore indicates a “wrong” direction of renewal and growth of cells. This could be the result of a loss of the regeneration balance resulting from obesity-induced environment impacting the regeneration or the cancer, depending on the situation, as illustrated by the links between the liver cancer progression and an abnormal microenvironment [51] or the effect of neurostimulation in both cancer and regeneration contexts, respectively [52]. Thus, understanding key differences between the physiological regeneration process and obesity-induced regeneration impairments combined with an understanding of obesity-associated cancer growth could contribute to a better understanding of carcinogenesis pathways.

Looking into subcellular and molecular pathways allows one to identify more mechanistic links between obesity and its negative impacts on regeneration. These include a reduced level of growth factors [53], altered DNA repair [54], induced DNA damage [55] and epigenetic changes [56]. This could explain the other important facts supporting the negative impacts obesity has on regeneration: reduced healing ability associated with obesity, as illustrated by the example of the slow bone fracture healing in obese mice [53], and increased risk of neurodegenerative diseases with obesity [57]. This shows the need to take particular care of obese patients recovering from diseases or conditions requiring regeneration, such as burns, radiotherapy, and leukemia, because of compromised regeneration ability.

Since stem cells are suggested to manage obesity [6,58,59], the negative environment generated by obesity could limit the efficacy of such an approach and therefore would first, as a preliminary step, reduce obesity (by diet, exercise or pharmacological tools) prior to stem cell administration, similarly to patients being prepared for bariatric surgery, in order to improve the biological environment receiving the stem cells and therefore optimize the therapeutic outcomes. In addition, since the negative impacts obesity has on regeneration are worsened by factors such as age [3], stem cell application to manage obesity should consider such factors towards more personalized stem cells-based therapies. Moreover, it is important to highlight the need for further studies to understand molecular players and allow one to define personalized treatments. The diversity of the available animal models of obesity could lead to differences in the results of obesity–regeneration interaction investigations. For instance, the use of diet-induced obesity animal models [60] would mimic obesity’s general effects, whereas the use of more specific models such as db/db or ob/ob animal models [61] would generate data closer to the effects of leptin in the obesity context.

The effects that an obesity-induced environment has on diverse regeneration patterns at different levels impact the development of different tissues as well as related metabolic, biochemical and physiological functions. Such impaired regeneration will have clinical, therapeutic and research applications and implications.
